# Genomic Characterization and Transcriptomic Analysis of the Phycobilisome Linker Proteins Family in *Pyropia haitanensis*

**DOI:** 10.3390/ijms27104408

**Published:** 2026-05-15

**Authors:** Fei Li, Haotian Wang, Yuqing Chen, Lanqi Yang, Peng Zhang, Shanshan Zhu

**Affiliations:** 1 State Key Laboratory for Managing Biotic and Chemical Threats to the Quality and Safety of Agroproducts, Ningbo University, Ningbo 315832, China; 2 Key Laboratory of Marine Biology Genetics and Breeding, Zhejiang Mariculture Research Institute, Wenzhou 316000, China

**Keywords:** *Pyropia haitanensis*, phycobilisome linker proteins, gene family evolution, functional differentiation

## Abstract

Phycobiliprotein linker polypeptides (PBLPs) are essential structural components of phycobilisomes (PBS), yet their composition, evolutionary trajectories, and regulatory functions in *Pyropia haitanensis* remain poorly understood. Here, we performed the first genome-wide identification and functional characterization of PBLPs in *P. haitanensis*. Nineteen *PBLP* genes were identified and classified into three subfamilies (LR, LRC, LC), exhibiting substantial physicochemical diversity and distinct gene structures. Phylogenetic and synteny analyses revealed extensive paralogous diversification driven primarily by dispersed duplication, with most duplicated pairs under strong purifying selection. Notably, the LCM subfamily was absent in *P. haitanensis* and *P. yezoensis*, suggesting lineage-specific gene loss and potential neofunctionalization of LR/LRC members. Transcriptome profiling demonstrated pronounced expression divergence between the wild-type (ZD) and red pigment mutant (RED) strains, with six *PBLP* genes showing significant differential expression validated by qRT-PCR. Under five irradiance levels, *PBLP* genes displayed distinct light-responsive transcriptional patterns. Mantel tests further revealed strong associations between PBLP expression and phycobiliprotein contents, photosynthetic pigments, and chlorophyll fluorescence parameters, indicating functional specialization within the family. Overall, this study provides comprehensive insights into the evolution, expression dynamics, and regulatory potential of PBLPs in *P. haitanensis*, highlighting their central roles in PBS assembly, pigment metabolism, and photophysiological acclimation. These findings establish a foundation for elucidating PBS regulatory mechanisms and improving pigment-related traits in economically important red algae.

## 1. Introduction

Phycobilisomes (PBS) are large, water-soluble light-harvesting complexes unique to cyanobacteria and red algae, serving as core functional units that enable these photosynthetic organisms to adapt to low-light environments and efficiently utilize blue-green light (450–650 nm) [1]. Situated on the stromal side of thylakoid membranes, PBS form functional complexes with photosystem II (PSII) and photosystem I (PSI) via specific anchor proteins, supporting rapid light capture and directional energy transfer. This process directly determines photosynthetic rate and biomass accumulation efficiency, and is fundamental to algal growth and development [2,3]. The structural stability and functional integrity of PBS rely on the coordinated action of two core components: phycobiliproteins (PBPs), which account for 85% of PBS mass and are responsible for light absorption, and phycobilisome linker proteins (PBLPs), which constitute only 15% of PBS mass and generally lack chromophores [4,5]. Despite their low abundance, PBLPs act as the structural backbone of PBS by mediating the assembly and arrangement of PBPs, maintaining supramolecular stability, and regulating energy transfer efficiency [5,6].

PBLPs are classified into four main categories based on their topology and function: rod linkers (LR), rod–core linkers (LRC), core linkers (LC), and core–membrane linkers (LCM) [6]. LR proteins contribute to the stabilization and structural organization of phycobiliproteins within rod elements. LRCs mediate the precise attachment of peripheral rods to the PBS core. LC proteins, which are characterized by the conserved Pfam01383 domain, play a critical role in guiding the assembly of core subunits. LCM proteins typically contain an α-helical domain along with Pfam00427 repeats, and serve to anchor the PBS core to thylakoid membranes, thereby promoting efficient energy transfer [7]. Recent studies have revealed that PBLPs function far beyond structural support. In the thylakoid-less cyanobacterium *Gloeobacter violaceus*, LRC proteins interact with α-APC through conserved α-helical loops, enabling light-dependent regulation of rod length [8]. In *Synechococcus* sp., the LCM protein LcpA acts as a molecular adhesive that connects PBS core proteins ApcB and ApcF to the CP47 subunit of PSII. The absence of LcpA abolishes photosynthetic oxygen evolution under green light, underscoring its critical role in energy transduction [9]. In the red alga *Porphyridium purpureum*, functional impairment of linker polypeptides has been shown to result not only in a significant decline in photosynthetic efficiency but also alter pigment phenotypes [8]. Moreover, under stress conditions such as high light intensity, elevated temperature, or nitrogen deprivation, PBLPs act as a regulatory hub for environmental adaptation by triggering complementary chromatic adaptation and remodeling PBS structure, thereby optimizing light harvesting and alleviating photodamage [10,11]. Collectively, these studies demonstrate that the molecular characteristics and regulatory mechanisms of PBLPs are closely linked to photosynthetic function, pigmentation, and stress tolerance. However, given the complexity of this gene family, genome-wide systematic characterization is still required across a broader range of algal species [12].

*P. haitanensis* is a dominant cultivated red alga along the southeastern coast of China, with high economic and ecological significance [13]. Its aquaculture yield and quality—including thallus color and thickness—are directly determined by photosynthetic efficiency, which is closely related to PBS functionality [14,15,16]. Previous studies have confirmed that the contents of PBPs (PE, PC, APC) in *P. haitanensis* are key determinants of photosynthetic rate, pigment phenotype (e.g., redness intensity), and commercial value [15]. However, the regulatory mechanisms of PBLPs—the essential scaffolds for PBP assembly and PBS structural maintenance—in the photosynthetic system and pigment formation of *P. haitanensis* remain largely unexplored. Specifically, PBLP structural stability directly influences PBS assembly efficiency and energy transfer efficiency, thereby modulating photosynthetic performance and biomass accumulation—core factors underlying yield. Furthermore, whether PBLPs functional divergence contributes to pigment biosynthesis and regulates thallus color phenotype— a key quality trait—has not been addressed. Despite the recognized central role of PBLPs in photosynthetic systems, our understanding of the *PBLP* gene family in *P. haitanensis* is limited: its genomic composition, evolutionary features, expression regulation, and molecular links to photosynthetic efficiency and pigment phenotype have not been systematically characterized.

To address these gaps, the present study performed the first comprehensive genome-wide analysis of the *PBLPs* gene family in *P. haitanensis* using a newly assembled telomere-to-telomere (T2T) genome. We systematically identified all PBLP family members and characterized their physicochemical properties, conserved domains, and gene structures. Phylogenetic and collinearity analyses were conducted to reveal evolutionary patterns and functional divergence. Promoter cis-element prediction was applied to elucidate the regulatory basis underlying responses to light, hormone, and stress, providing a theoretical framework for understanding adaptation to dynamic intertidal environments. Furthermore, by examining PBLP expression patterns in both the pigment mutant (RED) and wild-type (ZD) strains, as well as across a gradient of light intensities, we characterized the transcriptional responsiveness of the PBLP family under genetic and environmental variation. Based on the light-intensity experiment, Mantel tests were then applied to establish correlations between PBLP expression profiles and key physiological traits. Overall, this work elucidates the evolutionary divergence and structural-functional characteristics of the *PBLPs* gene family in *P. haitanensis*, providing a robust phenotypic and multi-omics foundation for molecular breeding programs.

## 2. Results

### 2.1. Identification and Physicochemical Characterization of the P. haitanensis PBLP Gene Family

Through a combined BLAST and HMMER search, we identified 19 complete genes belonging to the PBLPs family in the *P. haitanensis* (Table 1). These genes show significant variability in their physicochemical properties. The encoded proteins ranged in length from 131 to 3974 amino acids, with molecular weights varying from 13.98 to 400.48 kDa. *PhaLC* was the shortest protein, while *PhaLRγ7* was the longest. The theoretical isoelectric points (pI) spanned from 4.74 to 11.36, with the majority (89.47%) being basic (pI > 7); *PhaLRγ7* and *PhaLRC3* were the only acidic members (pI = 4.74, pI = 5.23). Despite the observed variability, all proteins were predicted to be unstable, as their calculated instability index (II) values ranged from 62.41 to 108.09, all of which are above the standard threshold of 40.

### 2.2. Phylogenetic Analysis and Classification of the PBLP Gene Family Members in P. haitanensis

**Figure 1:**
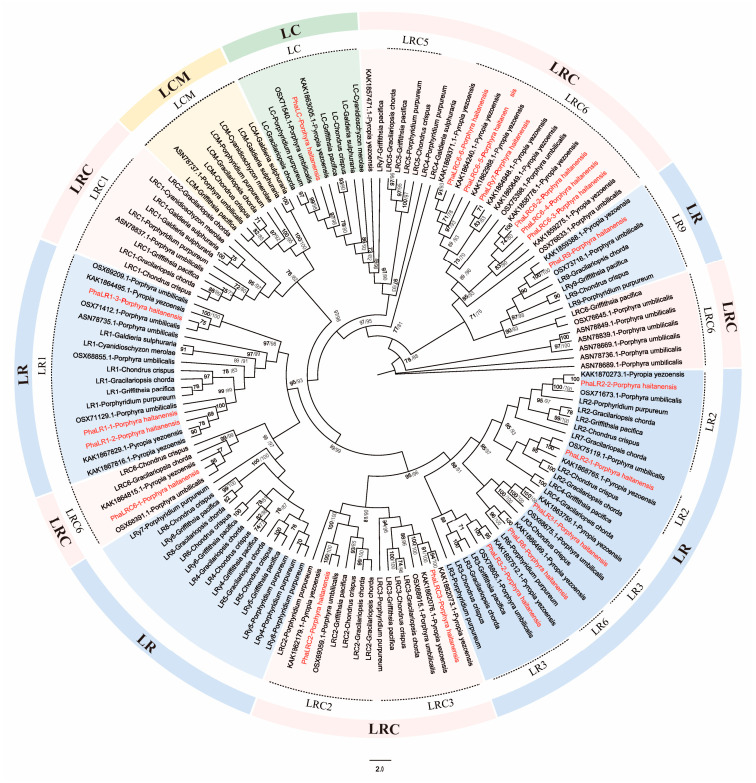
Phylogenetic tree analysis of PBLPs in *P. haitanensis* and other red algae. The Phylogenetic tree was constructed using IQ-TREE based on PBLP sequences. The analysis included sequences from *P. haitanensis*, *P. yezoensis*, *P. umbilicalis*, *C. crispus*, *G. chorda*, *C. merolae*, *G. sulphuraria*, *G. pacifica* and *P. purpureum*. Bootstrap support values (from 100 replicates) are shown at the nodes. The major subfamilies (e.g., LR, LRC, and LC) are delineated by distinct background colors. PBLPs from *P. haitanensis* are highlighted in red.

Based on an integrative analysis combining phylogenetic clustering and conserved domain architecture, the *P. haitanensis* PBLPs family members were classified into three subfamilies: LR, LRC, and LC (Figure 1). Phylogenetic reconstruction revealed that these subtypes do not form monophyletic clusters but are phylogenetically dispersed. This pattern suggests that the family may have undergone multiple early functional divergences. Among the identified proteins, LR had the most members (10), followed by LRC (8), while LC had only one member. Notably, the LR and LRC genes grouped into seven distinct clusters, a pattern consistent with observations in other red algae, supporting the hypothesis of functional specialization within homologous linker families [17]. Within the LRC subfamily, the LRC6 subtype showed a further split into two distinct clades located at different nodes of the phylogenetic tree. Additionally, the PBLPs from *P. haitanensis* showed close phylogenetic relationships with their orthologs in *P. yezoensis* and *P. umbilicalis*, providing strong evidence for the functional conservation of these proteins within the Rhodophyta.

### 2.3. Chromosomal Localization and Collinearity Patterns

**Figure 2:**
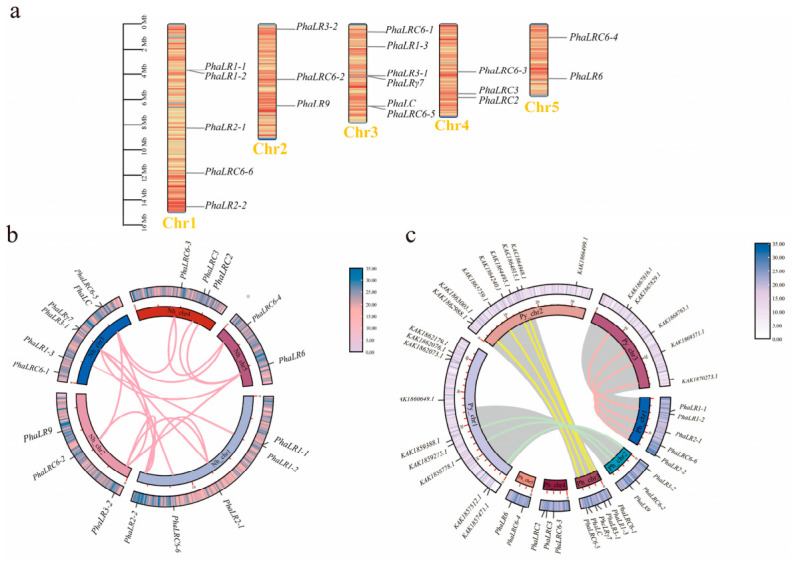
Chromosome mapping and colinearity analysis of the *P. haitanensis PBLPs* gene family. (**a**) Chromosomal localization of *PBLPs* genes. The positions of the *PBLPs* genes are marked on the *P. haitanensis* chromosomes; Different colors on the chromosomes indicate gene density. (**b**) Duplicate gene pairs in *P. haitanensis*. The inner ring and outer ring of the circular graph both represent *P. haitanensis* chromosomes, where the inner ring denotes the chromosomes themselves and the outer ring indicates gene density. Pink lines connect the 19 duplicated *PBLPs* gene pairs identified within these regions; (**c**) Inter-genomic synteny analysis of *PBLPs* genes between *P. haitanensis* and *P. yezoensis*. The inner ring and outer ring of the circular graph both represent chromosomes (inner ring: chromosomes; outer ring: gene density) of the two species, respectively. Gray ribbons represent syntenic blocks between the two species. Red lines, green lines, and yellow lines connect orthologous PBLPs gene pairs located on different chromosome pairs, respectively.

Chromosomal localization analysis showed that the *P. haitanensis PBLPs* genes are randomly spread across all five chromosomes (Figure 2a). Chromosome 1 (Chr1), the longest chromosome, contained five genes, whereas the shortest, Chr5, had two. Notably, Chr3, an intermediate-length chromosome, possessed the highest number of genes at six. Inter-species collinearity analysis identified 11 orthologous gene pairs between *P. haitanensis* and *P. yezoensis*, both from the Bangiaceae (Figure 2c), underscoring their phylogenetic proximity and highlighting the essential, conserved role of PBLPs in phycobilisome structure and energy transfer. Within the *P. haitanensis* genome, 19 duplicate gene pairs were found, all categorized as dispersed duplicates (Figure 2b; Table S2). Some of these duplicates, such as the paralogs *PhaLR1-1*, *PhaLR1-2*, and *PhaLR1-3*, are located on different chromosomes (Chr1 and Chr3) (Table S2). *K*a/*K*s analysis revealed that most duplicate gene pairs (18/19) had a *K*a/*K*s ratio less than 1, indicating strong purifying selection and suggesting their functions remained highly conserved after duplication (Table S2). Notably, the *K*a/*K*s ratio for *PhaLR2-1* and *PhaLR2-2* slightly exceeded 1 (1.041), suggesting this gene pair may have undergone relaxation or positive selection, warranting further investigation into their functional divergence.

### 2.4. Conserved Motifs, Gene Structures, and Protein Domains

**Figure 3:**
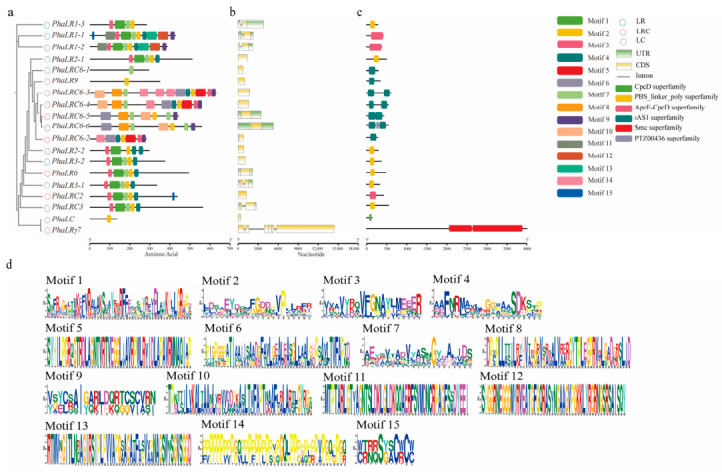
Functional domains of the *P. haitanensis PBLPs* gene family. (**a**) Phylogenetic analysis and conserved motifs. Unrooted phylogenetic tree of 19 PBLPs based on full-length protein sequences. Subfamilies are distinguished by purple, pink, and blue circles. The distribution of 15 conserved motifs identified by MEME is shown on the right; (**b**) Gene structure organization. Yellow boxes, green boxes, and black lines represent coding sequences (CDS), untranslated regions (UTRs), and introns, respectively. The scale bar is in kilobases (kb); (**c**) Conserved domain architecture. Domains annotated using the NCBI-CDD are represented by colored boxes; (**d**) Sequence logos of conserved motifs. The overall stack height indicates sequence conservation (in bits), while the height of each letter reflects the relative frequency of an amino acid.

Analyzing conserved motifs and gene structures is essential for understanding protein functional differentiation. We identified 15 motifs within the *PBLPs* gene family (Figure 3a,d). Motif 2 was present in all members, indicating it constitutes a core functional region. Subfamily-specific motifs were also observed: the LR subfamily contained the unique Motif 1, potentially involved in rod-like structure assembly, while the LRC subfamily possessed five unique motifs (5, 6, 8, 10, 14) that may form core-rod connections. Interestingly, *PhaLRγ7* lacked all typical motifs, suggesting it might be a novel functional subtype (Figure 3a,d).

Gene structure analysis (Figure 3b) revealed high conservation within the PBLPs gene family, characterized by a low intron count. Specifically, 13 genes were intronless, five contained a single intron, and only one gene possessed three introns. The LR and LRC subfamilies generally had simpler structures (zero or one intron), whereas the single LC member had a more complex structure with three introns, reflecting differing evolutionary constraints and potentially correlating with functional specialization.

Conserved domain analysis further highlighted subfamily differentiation (Figure 3c). The LR subfamily displayed the greatest diversity, featuring shared and unique domains such as PBS linker poly, ApcE-CpcD, and Smc superfamily domains. The LRC subfamily was distinguished by the unique presence of the PTZ00436 superfamily domain. In contrast, the LC subfamily had the simplest architecture, comprising only a CpcD superfamily domain. These distinct domain compositions likely underpin their specific functional roles within the phycobilisome.

### 2.5. Protein Structure Prediction

**Figure 4:**
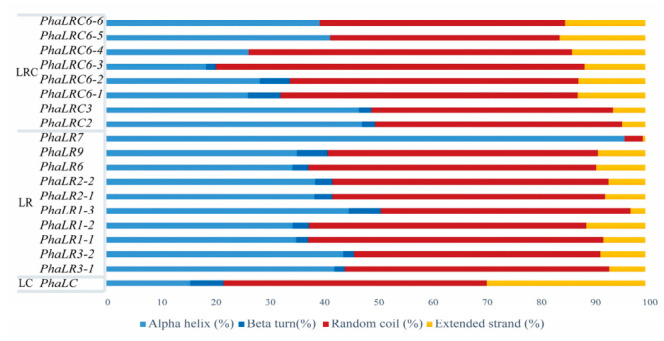
Predicted secondary and tertiary structures of *P. haitanensis* PBLPs. Secondary structure composition. The relative proportions of alpha-helices, extended strands, beta-turns, and random coils for each PBLPs protein, as predicted by SOPMA, are shown.

Secondary structure prediction based on the consensus of SOPMA and GOR4 algorithms for all PBLPs models, which had confidence scores exceeding 90%, showed a predominance of random coils (3.42%~68.38%) and α-helices (15.65%~96.14%) (Figure 4). A notable exception was *PhaLRγ7*, which was predicted to adopt an almost exclusively α-helical conformation (96.14%), hinting at a specialized function. Three-dimensional modeling via SWISS-MODEL and AlphaFold revealed complex spatial architectures incorporating various secondary structure elements (Figure S1). Each subfamily exhibited distinct spatial conformations, with conserved domains often forming a framework of parallel α-helices, a feature likely critical for phycobilisome assembly and stability.

### 2.6. Promoter Cis-Acting Elements Analysis

**Figure 5:**
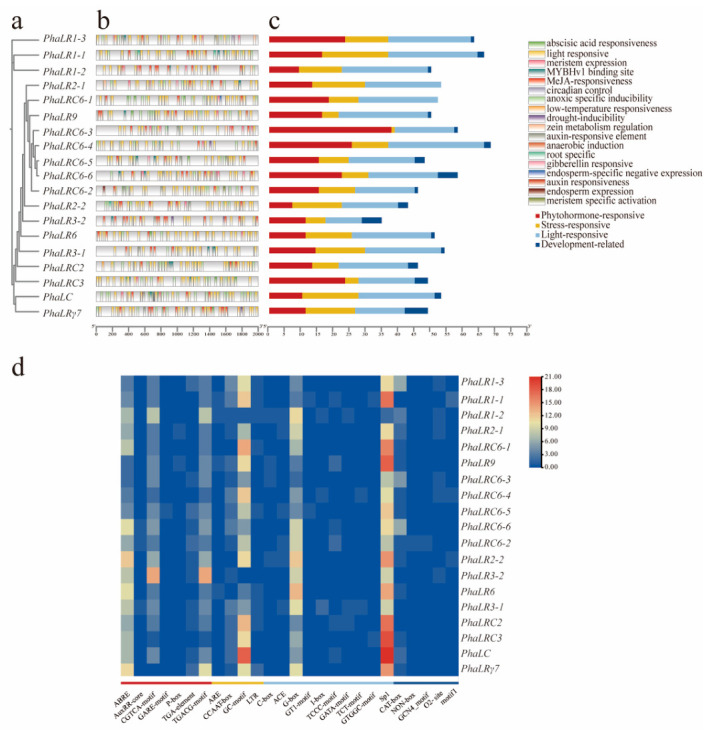
Cis-regulatory elements in the promoter region of the *PBLPs* gene in *P. haitanensis*. (**a**) Phylogenetic tree. The phylogenetic tree of PBLPs is provided for reference, organizing the display of promoter elements; (**b**) Schematic map of cis-acting elements. A diagram illustrating the distribution and number of various cis-acting elements identified within each PBLPs promoter. Elements are color-coded by their functional category; (**c**) Classification and counts of cis-acting elements. A stacked bar chart showing the total number of elements belonging to different functional categories (e.g., light, hormone, stress response) for each promoter. Different colors of the bars represent elements from different functional categories. (**d**) Heatmap of specific element abundance. A clustered heatmap visualizing the counts of key cis-acting elements across all promoter regions. The color intensity corresponds to the number of occurrences.

To elucidate the transcriptional regulatory mechanisms of the *P. haitanensis PBLPs* gene family, cis-acting elements were predicted in the promoter region spanning 2000 bp upstream of the transcription start site. A total of 33 cis-acting elements were identified, categorized into four major functional groups: 7 light-responsive elements, 7 hormone-responsive elements, 5 stress-responsive elements, and 14 development-related elements (Figure 5).

Among these, the G-box and Sp1 light-responsive elements were present in the promoters of all genes, indicating that light signals strictly regulate gene expression in this family and may be widely involved in light-induced transcription processes. Abscisic acid response elements (ABRE) and methyl jasmonate response elements (CGTCA/TGACG motif) were the most common within the hormone response category. Methyl jasmonate response elements comprised 23.7% of all hormone response elements, absent only in the specialized gene *PhaLRγ7*. In the stress response module, hypoxia response elements (GC-motif) appeared in 89.5% of genes, likely reflecting adaptation to the periodic hypoxic stress in the intertidal zone environment of *P. haitanensis*. Development-related cis-acting elements showed few meristem-specific expression elements, and while CAT-box elements were dominant but not universal. These findings suggest that the *PBLPs* gene family integrates light signals, hormonal cues, and abiotic stress responses to regulate growth and adaptation.

### 2.7. Expression Patterns of PBLPs in the Pigment Mutant and qRT-PCR Validation

**Figure 6:**
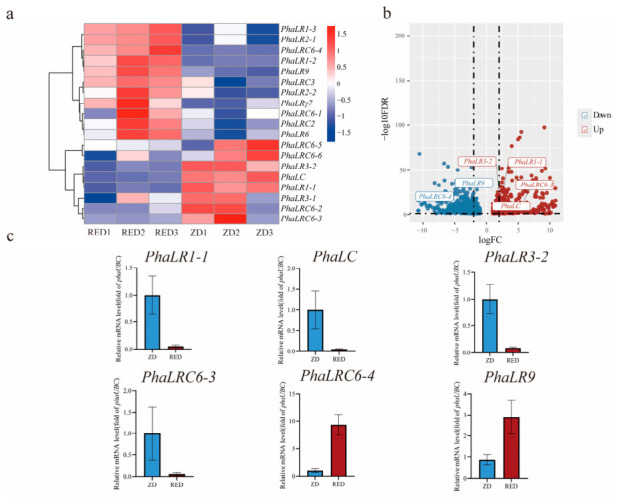
Expression analysis of the *PBLPs* gene family in the wild-type and RED mutant strains of *P. haitanensis*. (**a**) Expression heatmap of *PBLPs* genes. The heatmap displays the log2-transformed FPKM values for all *PBLPs* genes in wild-type and RED mutant strains. The color scale indicates the relative expression level from low (blue) to high (red); (**b**) Volcano plot of differentially expressed genes (DEGs). The plot visualizes the log2(FoldChange) versus the −log10(FDR) for all genes. Significant DEGs (FDR < 0.05, |log_2_FC| ≥ 1) are highlighted, with specific PBLP genes labeled. The dashed lines represent the thresholds for defining significant DEGs: the horizontal line indicates FDR = 0.05, and the vertical lines indicate log_2_FC = ±1; (**c**) qRT-PCR validation of selected *PBLP* genes. The bar charts illustrate the relative expression levels of selected DEGs in the RED mutant compared to the wild-type strain.

The red mutant (RED) of *P. haitanensis* exhibits a pronounced pigment phenotype, suggesting that alterations in phycobilisome structure or regulation may underlie its coloration (Figure S2). To determine whether PBLP gene expression contributes to this phenotype, we performed a comparative transcriptomic analysis between the wild-type (ZD) and RED strains. Among the 19 identified *PBLP* genes, 11 were upregulated and 8 were downregulated in the RED mutant relative to ZD (Figure 6a). Using a threshold of FDR < 0.05 and |log2FoldChange| ≥ 1, six genes exhibited significant differential expression, including two significantly upregulated and four significantly downregulated genes in the RED strain (Figure 6b).

To validate the reliability of the transcriptomic data, all six significantly differentially expressed PBLP genes were subjected to qRT-PCR analysis using three biological replicates. The quantitative results demonstrated that the transcript levels of *PhaLRC6-3*, *PhaLR1-1*, *PhaLC*, and *PhaLR3-2* were markedly reduced in the RED mutant compared with those in ZD, by approximately 17.5-, 18.8-, 20.0-, and 11.5-fold, respectively. Conversely, *PhaLRC6-4* and *PhaLR9* were significantly upregulated in the RED strain, showing approximately 9.5- and 3.3-fold increases, respectively (Figure 6c). These results confirm the robustness of the transcriptome profiling and support the involvement of PBLP expression variation in pigment differentiation between the two strains.

### 2.8. Expression Patterns of PBLP Genes Under Different Light Intensities and Trait Association Analysis

**Figure 7:**
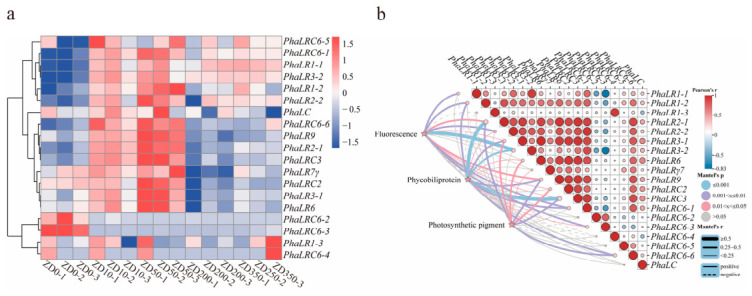
Expression patterns of the *PBLPs* gene family under different light intensities in *P. haitanensis*. (**a**) Expression heatmap of *PBLPs* genes. The heatmap displays the log2-transformed FPKM values for all *PBLPs* genes under different light treatments. The color scale indicates the relative expression level from low (blue) to high (red); (**b**) Mantel test and Pearson correlation analysis. The analysis shows the relationships between *PBLPs* gene expression and major physiological traits, including photosynthetic pigments, chlorophyll fluorescence parameters, and phycobiliprotein components, under different light treatments. Mantel analysis was performed only for the light-treatment group. Five-pointed stars represent the three physiological trait categories. The edge width corresponds to Mantel’s r, indicating the correlation strength, and the edge color denotes statistical significance (Mantel’s *p*), with pink lines indicating significant correlations (0.01 < *p* ≤ 0.05) and gray lines indicating non-significant correlations (*p* > 0.05). Solid and dashed lines represent positive and negative correlations, respectively. The triangular matrix displays pairwise Pearson’s correlation coefficients among individual *PBLPs* genes, with circle colors ranging from blue (negative correlation) to red (positive correlation), and circle size corresponding to the magnitude of the correlation coefficient (larger circles indicate stronger correlations).

To further elucidate the relationships between *PBLPs* gene expression and agronomic traits, we performed transcriptome sequencing and physiological measurements on wild-type *P. haitanensis* cultured under five light intensities. As shown in Figure S3, photosynthetic pigments (Chl a, carotenoids) and phycobiliproteins (PE, PC, APC, total PBP) displayed a pronounced increase–decrease pattern, peaking at 10 μmol·m^−2^·s^−1^ and declining thereafter. Fluorescence parameters changed more moderately: Fv/Fm, Φ(II), and qP decreased with increasing irradiance, whereas NPQ increased. Across irradiance gradients, PBLPs exhibited distinct expression patterns (Figure 7a), highlighting substantial variation in light-responsive behavior. Mantel test analysis further revealed strong and trait-specific associations between PBLP expression profiles and physiological parameters (Figure 7b). Fluorescence parameters showed significant correlations with a broad set of *PBLP* genes, including *PhaLR1-1*, *PhaLR1-2*, *PhaLR2-1*, *PhaLR2-2*, *PhaLR3-1*, *PhaLR3-2*, *PhaLR9*, *PhaLRC2*, *PhaLRC3*, *PhaLRC6-1*, and *PhaLRC6-3*, with *PhaLR3-2* exhibiting the strongest association (Mantel *r* > 0.5, *p* < 0.001). Phycobiliprotein contents were significantly associated with *PhaLR1-2*, *PhaLR2-1*, *PhaLR2-2*, *PhaLR3-1*, *PhaLR3-2*, *PhaLR6*, *PhaLR9*, *PhaLRC3*, *PhaLRC6-1*, *PhaLRC6-3*, and *PhaLRC6-6*, among which *PhaLR2-1* and *PhaLRC3* genes showed the strongest correlations (Mantel *r* > 0.5, *p* < 0.001). Photosynthetic pigments were primarily correlated with *PhaLR2-1*, *PhaLR3-1*, *PhaLR6*, *PhaLRγ7*, *PhaLR9*, *PhaLRC2*, *PhaLRC3*, and *PhaLRC6-6*, indicating a partially overlapping but distinct regulatory module compared with phycobiliproteins. Collectively, these results demonstrate that different subsets of PBLP genes are tightly linked to pigment accumulation, phycobiliprotein synthesis, and chlorophyll fluorescence responses, highlighting functional diversification within the PBLP family and underscoring their potential roles in coordinating light harvesting and photophysiological acclimation in *P. haitanensis*.

## 3. Discussion

PBLPs are essential structural components that determine the assembly, stability, and energy transfer efficiency of phycobilisomes (PBS), thereby directly affecting light-harvesting capacity and photosynthetic performance in red algae. While PBLPs have been characterized in several cyanobacterial and algal models [18,19], the composition, evolution, and functional roles of the *PBLPs* gene family in *P. haitanensis*—an economically significant intertidal red alga—remain largely unexplored. To address this knowledge gap, we performed the first genome-wide identification and systematic analysis of the *PBLPs* gene family in *P. haitanensis*, providing new insights into the functional divergence and adaptive regulation of this gene family.

A total of 19 non-redundant *PBLPs* genes were identified and classified into three subfamilies (LR, LRC, and LC). The gene number in *P. haitanensis* is intermediate among sequenced red algae, higher than that in *G. pacifica* and *P. purpureum* (17), but lower than in *P. yezoensis* (22) and *P. umbilicalis* (24) (Table S3). These proteins showed significant physicochemical diversity, as reflected in their isoelectric points (pI 4.74–11.36) and lengths (131–3974 amino acids). Such diversity likely supports the formation of specialized functional modules within the phycobiliprotein complex [20] and may be related to subcellular localization and light-harvesting specialization [3,7,21]. Gene structure analysis showed that the LR and LRC subfamilies generally have fewer introns and simpler gene structures, while the LC subfamily has a more complex intron-exon arrangement—these structural differences likely support functional differentiation and reflect long-term adaptive selection [20,22].

Phylogenetic analysis showed that *PBLPs* members did not form strictly monophyletic clades, with LR/LRC subfamily members dispersing across multiple branches—an evolutionary pattern consistent with that reported in other red algae [7]. Both subfamilies diverged into seven independent lineages, and LRC6 further split into two clades, indicating extensive paralogous diversification likely driven by gene duplication. In *P. haitanensis*, we identified 19 duplicated *PBLP* gene pairs, most of which originated from dispersed duplication—a mechanism known to promote sequence divergence and functional innovation [21,23]. Most duplicated pairs exhibited *K*a/*K*s ratios < 1, reflecting strong purifying selection that maintains core functional stability; however, the pair *PhaLR2-1*/*PhaLR2-2* showed an elevated ratio (≈1.04), indicating relaxed selective constraints that may promote adaptive evolution. Notably, the LCM subfamily was absent in both *P. haitanensis* and *P. yezoensis* (Table S3), representing a lineage-specific gene loss during *Pyropia* evolution. Since LCMs are traditionally considered critical for anchoring PBS to the thylakoid membrane [24], this raises the question of how PBS anchoring is achieved in these *Pyropia* species. We propose two non-mutually exclusive hypotheses: (1) Neofunctionalization of certain LR/LRC members compensates for LCM loss. PhaLRγ7 is a prime candidate, as it possesses a unique acidic pI and predicted all-α-helical structure—features that may enable membrane binding and replace LCM-mediated anchoring [24,25]. (2) The PBS core has evolved to interact directly with photosystem complexes [26], bypassing the need for a dedicated LCM linker. This adaptation could represent a specialized strategy to optimize energy transfer efficiency and genetic resilience under the fluctuating high-light conditions of the intertidal zone, reflecting a case of convergent evolution in Pyropia species [27].

Analysis of cis-acting elements in the promoter region highlighted the multifunctional role of this family in environmental responses and developmental regulation. Among plant hormone response elements, abscisic acid (ABA) response elements (ABRE) and methyl jasmonate (MeJA) response elements (CGTCA-motif, TGACG-motif) are predominant. Notably, all members except *PhaLRγ7* contain both ABRE and MeJA-related elements, suggesting the integration of ABA and MeJA signaling pathways in response to biotic stress [28]. Stress-responsive elements were also prominent, with the hypoxia-responsive GC-motif occurring in 89.5% of promoters, highlighting the importance of this gene family in adaptation to periodic hypoxia in the intertidal zone. Light-responsive elements, such as the G-box and Sp1, were universally present, indicating a conserved role in light perception and photosynthetic regulation [29]. Development-related elements like the CAT-box show limited distribution, implying regulation in specific tissues or developmental stages. These results demonstrate that PBLP genes are tightly regulated by external environmental signals, enabling dynamic remodeling of PBS structure to maintain photosynthetic homeostasis under changing conditions. Expression divergence between the RED mutant and wild type further supports functional specialization within the PBLP family. The RED mutant accumulates significantly higher phycobiliproteins and photosynthetic pigments (Figure S2), while maintaining comparable chlorophyll fluorescence parameters, indicating that its phenotype arises from pigment composition rather than impaired PSII function [4,30]. Correspondingly, transcriptome and qRT-PCR analyses (Figure 6) revealed that several PBLP genes essential for PBS structural organization—such as *PhaLRC6-3*, *PhaLR1-1*, *PhaLC*, and *PhaLR3-2*—were drastically downregulated in RED, suggesting disrupted linker–chromophore coordination that may alter PBS assembly or energy transfer routes, a mechanism also reported in structural studies of PBS linker proteins [31,32,33]. In contrast, the strong upregulation of *PhaLRC6-4* and *PhaLR9* may represent compensatory transcriptional responses aimed at stabilizing PBS function under altered pigment stoichiometry, consistent with regulatory adjustments observed in PBS-related networks in cyanobacteria and red algae [34,35]. Together, these findings demonstrate that PBLP expression variation is tightly coupled to pigment accumulation patterns and likely contributes to the distinct coloration and physiological characteristics of the RED mutant [3,36].

The light-responsive expression patterns of PBLP genes further underscore their central roles in regulating pigment metabolism and photophysiological acclimation [11]. Under five irradiance levels, photosynthetic pigments and phycobiliproteins exhibited a characteristic rise-and-fall pattern (Figure S3), peaking at moderate light, whereas chlorophyll fluorescence parameters changed more subtly [37,38]. Such differential light responses are consistent with the known plasticity of PBS components in intertidal red algae [33,39,40]. Correspondingly, PBLPs displayed diverse transcriptional responses across irradiance gradients (Figure 7a), indicating substantial variation in light-responsive behavior [41]. Mantel test analyses (Figure 7b) revealed strong and trait-specific associations between PBLP expression and pigment or fluorescence parameters, with genes such as *PhaLR3-2*, *PhaLR2-1*, and *PhaLRC3* showing particularly strong correlations (Mantel r > 0.5). These associations suggest that different subsets of PBLPs contribute to specialized functional modules—some linked to phycobiliprotein accumulation, others to chlorophyll-based pigmentation or photochemical efficiency—consistent with the modular organization of PBS linker proteins reported in previous studies [17,31]. This functional diversification likely enhances the ability of *P. haitanensis* to maintain photosynthetic homeostasis in the dynamic light environment of the intertidal zone, a key adaptive feature of red algae inhabiting fluctuating irradiance regimes [35,37]. In summary, our integrated genomic, evolutionary, and expression analyses reveal that the PBLP gene family in *P. haitanensis* exhibits substantial structural diversity, lineage-specific evolutionary patterns, and finely tuned transcriptional regulation. These features collectively support functional differentiation within the family and underscore its central roles in PBS assembly, pigment metabolism, and light-acclimation strategies. This work provides a comprehensive framework for understanding how PBLPs contribute to the adaptive success of *P. haitanensis* in fluctuating intertidal environments.

## 4. Materials and Methods

### 4.1. Identification and Physicochemical Characterization of the PBLPs in P. haitanensis

Members of the PBLP gene family in *P. haitanensis* were identified using a dual-strategy pipeline implemented in TBtools v1.108 [42], based on proteome sequences from a newly assembled telomere-to-telomere reference genome generated by our laboratory (unpublished). First, BLASTP searches were performed using experimentally validated PBLP sequences from *G. pacifica* [2] and *P. purpureum* [8] as queries, with an E-value threshold of 1 × 10^−5^. These two species were selected because they represent distinct red algal lineages and provide reliable reference sequences for comprehensive homolog retrieval. Second, Hidden Markov Models (HMMs) were built using TBtools v1.108-HMMER based on the PBLPs domain (PF00427 and PF01383) from the Pfam database, and HMMER v3.3 was employed to search the *P. haitanensis* protein database using an E-value cutoff of 1 × 10^−5^. To ensure high reliability, all candidate proteins were strictly verified for the presence of complete functional domains using both the NCBI Conserved Domain Database (CDD) (https://www.ncbi.nlm.nih.gov/Structure//cdd/wrpsb.cgi, accessed on 12 May 2025) and the Simple Modular Architecture Research Tool (SMART) database (https://smart.embl-heidelberg.de/, accessed on 31 January 2026). Furthermore, to exclude potential pseudogenes and verify transcriptional activity, RNA-seq data were analyzed, and candidates were further filtered using the criterion of FPKM > 1. Additionally, TBtools v1.108-Protein Paramter Calc was utilized to analyze physicochemical parameters, such as molecular weight and isoelectric point, for each member. This methodology was also applied to identify PBLP gene family members in the related species *P. yezoensis* (GenBank accession number: GCA_009829735.1) and *P. umbilicalis* (GenBank accession number: GCA_002049455.2).

### 4.2. Phylogenetic Analysis

To establish a robust phylogenetic framework for the classification of the PBLP gene family, a comprehensive phylogenetic analysis was performed using both Maximum Likelihood (ML) and Neighbor-Joining (NJ) methods. The analysis utilized linker polypeptide sequences from nine red algae species, including *P. haitanensis*, *P. yezoensis*, *P. umbilicalis*, *C. crispus*, *G. chorda*, *C. merolae*, *G. sulphuraria*, *P. purpureum*, and *G. pacifica* [2]. These species were selected for their comprehensive coverage of major Rhodophyta lineages (Bangiales, Florideophyceae, Cyanidiophyceae, Porphyridiophyceae), well-annotated genomes and characterized PBLPs sequences, as well as diverse ecological habitats and life forms, ensuring reliable and representative phylogenetic inference. Multiple sequence alignment was performed with TBtools v1.108-MUSCLE using default parameters, and the resulting alignment was subsequently refined by trimming poorly aligned regions and handling gap-rich sites using TBtools v1.108-TrimAL v1.4 (gappyout mode). The ML tree was constructed using TBtools v1.108-IQ-TREE, and the NJ tree was inferred using MEGA11. Statistical confidence for nodal support was evaluated through 1000 bootstrap replications for both ML and NJ analyses. Given the high topological consistency between the ML and NJ trees, the final phylogenetic tree is presented based on the ML topology, with bootstrap support values from both methods (ML/NJ) annotated on the corresponding nodes.

### 4.3. Chromosomal Localisation and Collinearity Analysis

Chromosomal locations of the identified *P. haitanensis* PBLP genes were mapped based on the genome annotation file (GFF3 format) using TBtools v1.108. To identify conserved homologous genes between *P. haitanensis* and *P. yezoensis*, whole genome synteny analysis was conducted with TBtools v1.108-MCScanX with default parameters (e-value = 1 × 10^−5^, minimum matching genes ≥ 5). The synteny results were visualized using TBtools v1.108-Circos. Gene duplication events within the *P. haitanensis* PBLPs family were analyzed using DupGen_finder [23] and the duplication types of each identified duplicated gene pair were explicitly classified into five categories: whole-genome duplication (WGD), tandem duplication, proximal duplication, transposed duplication, and dispersed duplication. To evaluate their evolutionary rates and selective pressures acting on the duplicated gene pairs, the rate ratio of non-synonymous to synonymous substitution (*K*a/*K*s) was calculated using KaKs_Calculator v3.0, adopting a length-normalized method [43].

### 4.4. Motif Identification and Gene Structure Analysis

Conserved protein motifs were identified using the MEME Suite (https://meme-suite.org/meme/, accessed on 16 May 2025) [44]. The parameters were set to a maximum of 15 motifs, with motif widths ranging from 6 to 50 amino acids. Gene structures (exon-intron organization) were analyzed using TBtools v1.108 in conjunction with genomic annotation data. Protein domains were annotated via the NCBI Conserved Domains Database and the resulting domain architectures were visualized using TBtools v1.108.

### 4.5. Promoter Cis-Acting Element Analysis

A 2000 bp sequence upstream of each gene’s transcription start site (TSS) was extracted as the putative promoter region. Cis-acting elements within these promoter sequences were identified using the PlantCARE database (https://bioinformatics.psb.ugent.be/webtools/plantcare/html/, accessed on 12 June 2025) [13]. Key elements involved in light response, hormone response and stress response were selected, and their distribution was visualized as a clustered heatmap using the pheatmap package in RStudio v4.4.1.

### 4.6. Two-/Three-Dimensional Structure Prediction

Protein secondary structures were predicted using the SOPMA online analysis tool (https://npsa.lyon.inserm.fr/cgi-bin/npsa_automat.pl?page=/NPSA/npsa_sopma.html, accessed on 26 July 2025) and GOR4 (https://npsa.lyon.inserm.fr/cgi-bin/npsa_automat.pl?page=/NPSA/npsa_gor4.html, accessed on 30 January 2026). Pearson correlation analysis confirmed high consistency between the two methods, with correlation coefficients (r) for all structural elements exceeding 0.75. Consequently, the simple average of the percentage data from both tools was calculated and used as the final secondary structure prediction for the target proteins. For three-dimensional structure prediction, SWISS-MODEL [45] (https://swissmodel.expasy.org/, accessed on 26 July 2025) was employed to select optimal templates with sequence identity greater than 30%, and models with a Global Model Quality Estimation (GMQE) score ≥ 0.5 and QMEANDisCo Global score ≥ 0.6 were considered reliable. For proteins with low sequence homology, ab initio modeling was performed using AlphaFold (https://alphafoldserver.com, accessed on 30 January 2026) to ensure structural accuracy.

### 4.7. Experimental Strains and Treatment Design

Healthy thalli of *P. haitanensis* (ZD and RED) were obtained from the Seaweed Germplasm Bank of Ningbo University. All thalli were maintained in aerated culture using filtered seawater enriched with Ningbo University No. 3 nutrient solution, which was refreshed every two days. Two independent experimental modules were conducted: (1) Strain Comparison: The ZD and RED strains were cultured at 21 °C under a 12 h light:12 h dark photoperiod with a light intensity of 40 μmol/(m^2^·s) to investigate physiological and transcriptomic differences. (2) Light-intensity Response: Healthy ZD thalli were cultured at 21 °C under a 12 h light:12 h dark photoperiod and exposed to five light intensities: 0, 10, 50, 200, and 350 μmol/(m^2^·s). After 48 h of treatment, the thalli were harvested, gently blotted dry, and immediately snap-frozen in liquid nitrogen. All samples were collected with three biological replicates per treatment and stored at −80 °C for subsequent physiological and transcriptomic analyses.

### 4.8. Determination of Photosynthetic Pigments and Chlorophyll Fluorescence Parameters

The contents of phycobiliproteins and photosynthetic pigments from both experimental modules were determined based on the methods described by Zhao [46] and Moran [47], with slight modifications. Healthy thalli of the two strains (ZD and RED) were selected with three biological replicates, weighed, flash-frozen, and ground into fine powder. Under dark and ice-bath conditions, 1.6 mL of phosphate-buffered saline (PBS, pH 5.5) (10× Phosphate Buffered Saline (PBS) Solution, Beijing Solarbio Science & Technology Co., Ltd., Beijing, China) was added to the samples. The mixtures were sonicated for 2 min and subjected to a freeze–thaw cycle (−20 °C for 2 h, followed by thawing at 4 °C for 1 h) to sufficiently extract phycobiliproteins. After centrifugation (3000× *g*, 4 °C, 5 min), 100 μL of the supernatant was transferred to a 96-well plate, and the absorbances at 498, 614, and 651 nm were measured using a microplate reader (Varioskan Flash Multimode Reader, Thermo Fisher Scientific, Vantaa, Finland). Subsequently, the remaining supernatant was discarded, and 2 mL of methanol (Methanol, AR grade, Sinopharm Chemical Reagent Co., Ltd., Shanghai, China) was added to the residual pellet. The mixture was incubated in the dark at 4 °C for 24 h to extract photosynthetic pigments. Following centrifugation (5000 rpm, 4 °C, 5 min), the absorbances of the supernatant were recorded at 480, 510, 652, 665, and 750 nm. The contents of phycoerythrin, phycocyanin, allophycocyanin, chlorophyll a, and carotenoids were then calculated using standard equations. Additionally, for the determination of chlorophyll fluorescence parameters, healthy thalli were dark-adapted for 15 min. Parameters of the two strains, including minimum fluorescence, maximum fluorescence, maximum photochemical efficiency of PSII, effective photochemical efficiency of PSII, non-photochemical quenching, and photochemical quenching, were measured using the WATER-EDF1.5R Fluorescence Monitoring System (Heinz Walz GmbH, Effeltrich, Germany) with WinControl v3.0 [48]. Differences between ZD and RED strains were assessed using independent-samples *t*-tests, while differences among light-intensity treatments were analyzed via one-way ANOVA followed by Duncan’s test, with a *p*-value < 0.05 considered statistically significant.

### 4.9. Expression Patterns, Trait Association Analysis, and qRT-PCR Validation

Expression patterns of *PBLPs* genes under different strains and light conditions were analyzed using RNA-seq data. The preserved thalli samples from both modules were sent to Benagen Technology (Wuhan Benagen Technology Company Limited, Wuhan, China) for transcriptome sequencing, with three biological replicates set for each treatment. An expression heatmap was generated in R Studio v4.4.1 based on log2-transformed FPKM values to visualize the expression profiles across strains and light intensities. Differentially expressed genes (DEGs) were identified using DESeq2 with the thresholds of FDR < 0.05 and |log2FoldChange| ≥ 1, and were visualized using a volcano plot in R. To validate the transcriptomic results, six PBLP genes showing significant differential expression between the ZD and RED strains were selected for qRT-PCR analysis. Gene-specific primers were designed using Primer Premier v5.0 (Table S1). Total RNA was isolated from preserved thalli using HiPure Plant RNA Mini Kit (Magen, Guangzhou, China) according to the manufacturer’s protocols. The obtained total RNA was then reverse-transcribed into first-strand cDNA with genomic DNA removal using ABScript III RT Master Mix for qPCR with gDNA Remover (ABclonal, Wuhan, China). qRT-PCR reactions were performed on a QuantStudio™ 6 Flex Real-Time PCR System using SYBR Green Master Mix (2× Universal SYBR Green Fast qPCR Mix, ABclonal Technology Co., Ltd., Wuhan, Hubei, China). *PhUBC* was employed as the internal reference gene, consistent with its stable expression across *P. haitanensis* tissues and treatments [49]. Relative transcript levels were calculated using the 2^−ΔΔCt^ method, with three biological and three technical replicates included for each gene. Furthermore, specifically for the light-intensity response experiment, to investigate the correlations between gene expression profiles and the aforementioned physiological traits (including chlorophyll fluorescence parameters, phycobiliproteins, and photosynthetic pigments), a Mantel test was performed. Statistical analysis and corresponding visualization were conducted using the ChiPlot online platform (https://www.chiplot.online/, accessed on 18 April 2026) [50].

## 5. Conclusions

This study systematically analyzes the *PBLPs* gene family in *P. haitanensis*, revealing its structural diversity, evolutionary history, and regulatory complexity. The 19 identified PBLP genes exhibit clear subfamily differentiation, distinct gene architectures, and evidence of dispersed duplication as the major driver of expansion. The absence of the LCM subfamily in *Pyropia* species suggests a lineage-specific evolutionary event, potentially compensated by neofunctionalized LR/LRC members or alternative PBS–photosystem interactions. Expression analyses demonstrated that PBLP transcription is tightly linked to pigment composition and PBS functionality, with the RED mutant showing significant upregulation of key structural genes and corresponding increases in pigment accumulation. Light-intensity experiments further revealed diverse transcriptional responses and strong correlations between PBLP expression and physiological traits, indicating functional specialization in light harvesting and photoprotection. Together, these findings provide a comprehensive framework for understanding the molecular basis of PBS regulation in *P. haitanensis*. The identified candidate genes and regulatory modules offer valuable targets for future functional studies and molecular breeding aimed at improving pigment traits and stress resilience in economically important red algae.

## Figures and Tables

**Table 1:** Basic Information on Members of the *P. haitanensis PBLPs* Gene Family.

Gene ID	Classification	Gene Name	Number of Amino Acid	Molecular Weight	Theoretical pI	Instability Index	Aliphatic Index	Grand Average of Hydropathicity
*Pha00776.1*	LR1	*PhaLR1-1*	410	44,008.46	9.35	43.37	62.41	−0.33
*Pha00782.1*	LR1	*PhaLR1-2*	371	39,871.67	9.30	33.30	65.80	−0.32
*Pha05622.1*	LR1	*PhaLR1-3*	272	28,467.15	8.66	38.50	76.95	0.01
*Pha01713.1*	LR2	*PhaLR2-1*	490	52,071.83	9.49	35.09	69.67	−0.32
*Pha03088.1*	LR2	*PhaLR2-2*	285	29,351.26	8.49	30.80	79.86	0.18
*Pha06143.1*	LR3	*PhaLR3-1*	321	34,928.61	8.76	30.61	69.44	−0.30
*Pha03272.1*	LR3	*PhaLR3-2*	360	38,857.84	8.59	46.73	67.89	−0.29
*Pha09655.1*	LR6	*PhaLR6*	473	49,243.50	8.88	44.99	78.12	−0.06
*Pha06152.1*	LRγ7	*PhaLRγ7*	3974	400,480.06	4.74	30.69	86.65	−0.16
*Pha04661.1*	LR9	*PhaLR9*	336	34,630.53	9.28	40.42	84.64	0.08
*Pha08312.1*	LRC2	*PhaLRC2*	419	45,615.25	8.82	52.86	69.40	−0.32
*Pha08241.1*	LRC3	*PhaLRC3*	540	57,141.38	5.23	45.12	80.69	−0.19
*Pha05364.1*	LRC6	*PhaLRC6-1*	283	28,668.62	8.77	48.33	87.35	0.24
*Pha04182.1*	LRC6	*PhaLRC6-2*	270	27,515.29	10.88	48.22	104.15	0.38
*Pha07835.1*	LRC6	*PhaLRC6-3*	604	61,759.93	11.36	62.46	81.80	−0.12
*Pha08895.1*	LRC6	*PhaLRC6-4*	535	54,380.21	11.25	62.22	85.81	−0.06
*Pha06698.1*	LRC6	*PhaLRC6-5*	425	42,885.55	10.39	25.08	108.09	0.42
*Pha02415.1*	LRC6	*PhaLRC6-6*	535	53,381.76	10.01	26.01	99.83	0.50
*Pha06692.1*	LC	*PhaLC*	131	13,989.03	10.15	49.45	67.10	−0.20

## Data Availability

The original contributions presented in the study are included in the article/Supplementary Material, further inquiries can be directed to the corresponding authors.

## Supplementary Materials

The following supporting information can be downloaded at: https://www.mdpi.com/article/10.3390/ijms27104408/s1. Reference [49] is cited in the Supplementary Materials.

## Abbreviations

The following abbreviations are used in this manuscript:

PBS	Phycobilisomes
PBLPs	Phycobilisome Linker Proteins
PBPs	Phycobiliproteins
PSII	Photosystem II
PSI	Photosystem I
PE	Primarily phycoerythrin
PC	Phycobilisome Linker Proteins
APC	Allophycocyanin
LR	Rod linkers
LR	Rod–core linkers
LRC	Core linkers
LCM	Core–membrane linkers
Chl a	Chlorophyll a
Car	Carotenoids
Fo	Minimum fluorescence
Fm	Maximum fluorescence
Fv/Fm	Maximum photochemical efficiency of PSII
NPQ	Non-photochemical quenching
qP	Photochemical quenching
