# Supplementary material for: Genomic Characterization and Transcriptomic Analysis of the Phycobilisome Linker Proteins Family in Pyropia haitanensis
Source: Int J Mol Sci. 2026 May 15;27(10):4408. doi: 10.3390/ijms27104408 (PMC13208050; doi:10.3390/ijms27104408)
Supplement: Supplementary file 1 [file ijms-27-04408-s001.zip › ijms-4115773-supplementary.pdf]

Supplementary Materials

Table S1. Primers used for qRT-PCR analysis.

Gene Name	Forward primer	Reverse primer
PhUBC [1]	TCACAACGAGGATTTACCACC	GAGGAGCACCTTGGAAACG
PhaLRC6-3	TCAAGTCCAACGAGTACAAG	TACTCACCATCGTCCAGG
PhaLR1-1	GCGGACTATGAGGAGATTAT	TTGTACTCGTTGGACTTGAC
PhaLRC6-4	GTGCTGACGGTCTTTGC	TCAAAAAGTTGAAGATGGGC
PhaLR3-2	GTTTAAGCACCTACTGGGG	AAAGTCGTCACCAAACTTCT
PhaLC	CTCACCACCGCCTTTAC	GCTGCCATTCATGTCATAGT
PhaLR9	CATCCACGAGAACCTCAAG	CAAAGACCATAAAGTTGCCC

Table S2. List of dispersed duplicate gene pairs and their Ka/Ks values in *Pyropia haitanensis.*

Gene duplication	Duplicate 1	Location	Duplicate 2	Location	E-value	Ka/Ks
Dispersed duplication	*PhaLRC2*	Ph_chr4:5,840,684	*PhaLR6*	Ph_chr5:4,345,705	1.56×10^{-46}	0.94
Dispersed duplication	*PhaLRC3*	Ph_chr4:5,552,947	*PhaLR6*	Ph_chr5:4,345,705	1.90×10^{-37}	0.96
Dispersed duplication	*PhaLR3-1*	Ph_chr3:4,118,002	*PhaLR6*	Ph_chr5:4,345,705	9.98×10^{-44}	0.77
Dispersed duplication	*PhaLR3-2*	Ph_chr2:406,134	*PhaLR3-1*	Ph_chr3:4,118,002	2.25×10^{-42}	0.67
Dispersed duplication	*PhaLR1-1*	Ph_chr1:3,650,909	*PhaLR1-2*	Ph_chr1:3,680,496	0	0.37
Dispersed duplication	*PhaLR1-1*	Ph_chr1:3,650,909	*PhaLR1-3*	Ph_chr3:1,775,173	9.15×10^{-35}	0.64
Dispersed duplication	*PhaLR1-2*	Ph_chr1:3,680,496	*PhaLR1-3*	Ph_chr3:1,775,173	2.23×10^{-35}	0.59
Dispersed duplication	*PhaLR2-1*	Ph_chr1:8,279,251	*PhaLR2-2*	Ph_chr1:14,509,029	1.48×10^{-38}	1.04
Dispersed duplication	*PhaLR2-1*	Ph_chr1:8,279,251	*PhaLR3-2*	Ph_chr2:406,134	5.57×10^{-35}	0.99
Dispersed duplication	*PhaLR2-1*	Ph_chr1:8,279,251	*PhaLR3-1*	Ph_chr3:4,118,002	5.01×10^{-32}	0.75
Dispersed duplication	*PhaLR2-2*	Ph_chr1:14,509,029	*PhaLR3-2*	Ph_chr2:406,134	5.21×10^{-36}	0.76
Dispersed duplication	*PhaLR2-2*	Ph_chr1:14,509,029	*PhaLR3-1*	Ph_chr3:4,118,002	1.04×10^{-36}	0.89
Dispersed duplication	*PhaLR6*	Ph_chr5:4,345,705	*PhaLR3-2*	Ph_chr2:406,134	9.63×10^{-36}	0.80
Dispersed duplication	*PhaLRC6-2*	Ph_chr2:4,415,708	*PhaLRC6-5*	Ph_chr3:6,516,202	2.44×10^{-13}	0.65
Dispersed duplication	*PhaLRC6-3*	Ph_chr4:3,814,463	*PhaLRC6-5*	Ph_chr3:6,516,202	1.93×10^{-12}	0.68
Dispersed duplication	*PhaLRC6-4*	Ph_chr5:1,104,982	*PhaLRC6-5*	Ph_chr3:6,516,202	3.38×10^{-12}	0.71
Dispersed duplication	*PhaLRC6-4*	Ph_chr5:1,104,982	*PhaLRC6-3*	Ph_chr4:3,814,463	4.42×10^{-120}	0.23
Dispersed duplication	*PhaLRC6-6*	Ph_chr1:11,822,271	*PhaLRC6-2*	Ph_chr2:4,415,708	2.09×10^{-21}	0.68
Dispersed duplication	*PhaLRC6-6*	Ph_chr1:11,822,271	*PhaLRC6-5*	Ph_chr3:6,516,202	2.40 × 10−20	0.46

Table S3. Statistics of *PBLP* genes in selected Rhodophyte species.

Classification\ Species	*Pyropia haitanensis*	*Pyropia yezoensis*	*Porphyra umbilicalis*	*Griffithsia pacifica*	*Porphyridium purpureum*	*Chondrus crispus*	*Cyanidi-oschyzon merolae*	*Galdieria sulphuraria*	*Gracilariopsis chorda*
LC	1	1	1	1	0	1	1	1	1
LCM	0	0	1	1	1	1	1	2	1
LR	10	9	14	9	7	7	1	1	9
LRC	8	12	8	6	5	3	1	3	8

Table S4. Photosynthetic parameters of wild-type (ZD) and red mutant (RED) strains of *P. haitanensis.*

Strain	Replicate	Chl a $(mg \cdot g^{-1})$	Car $(mg \cdot g^{-1})$	PE $(mg \cdot g^{-1})$	PC $(mg \cdot g^{-1})$	APC $(mg \cdot g^{-1})$	F_v/F_m	Φ(II)	NPQ	qP
ZD	1	0.7803	0.7256	15.4887	10.8019	13.8706	0.5753	0.5050	0.1278	0.8644
ZD	2	0.9318	1.0017	34.2673	18.0527	32.8010	0.6153	0.5250	0.2078	0.8744
ZD	3	0.6603	0.6564	14.8963	11.4016	13.7696	0.6553	0.5450	0.2878	0.8844
	Mean ± SD	0.79 ± 0.14	0.79 ± 0.18	21.55 ± 11.02	13.42 ± 4.02	20.15 ± 10.96	0.62 ± 0.04	0.53 ± 0.02	0.21 ± 0.08	0.87 ± 0.01
RED	1	2.6290	1.4128	44.2773	18.0219	29.0757	0.6000	0.4678	0.2558	0.8140
RED	2	3.5797	2.2150	41.6192	17.4607	16.8505	0.6200	0.4878	0.2758	0.8240
RED	3	5.2305	3.3876	52.3446	23.1402	38.5871	0.6400	0.5078	0.2958	0.8340
	Mean ± SD	3.81 ± 1.32	2.34 ± 0.99	46.08 ± 5.59	19.54 ± 3.13	28.17 ± 10.90	0.62 ± 0.02	0.49 ± 0.02	0.28 ± 0.02	0.82 ± 0.01

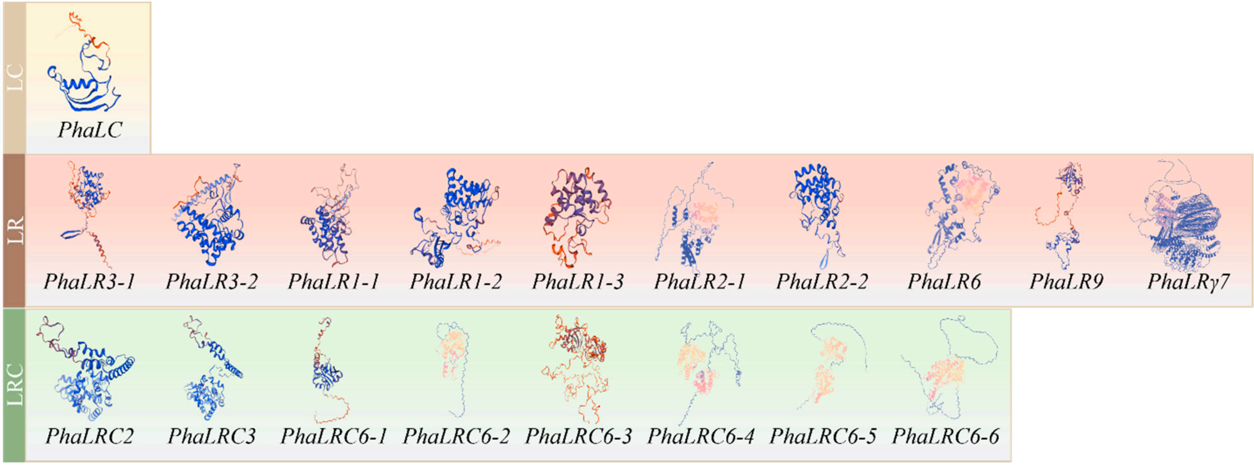

Figure S1. Tertiary structures of *P. haitanensis* PBLP proteins. Predicted tertiary structures. Homology-based 3D models of representative PBLP proteins gener-ated using the SWISS-MODEL server.

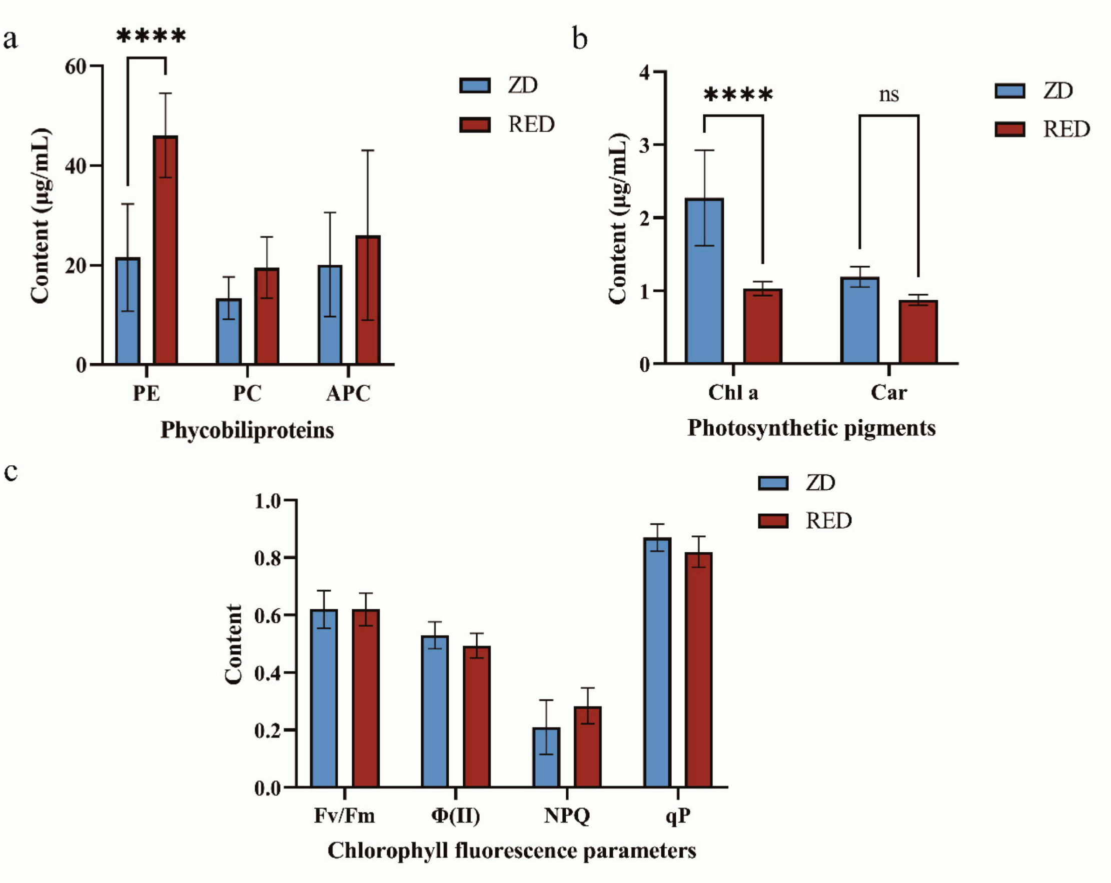

Figure S2. Physiological and phenotypic comparisons between the wild-type and red mutant strains. (**a**) Phycobiliprotein contents, including phycoerythrin, phycocyanin and allophycocyanin. (**b**) Photosynthetic pigment contents, including chlorophyll a and carotenoids. (**c**) Chlorophyll fluorescence parameters, including the maximum quantum yield of PSII, effective quantum yield of PSII, non-photochemical quenching and photochemical quenching. Asterisks indicate statistically significant differences between the ZD and RED strains based on Student's *t*-test (*** $p < 0.001$, **** $p < 0.0001$). Absence of asterisks indicates no significant difference.

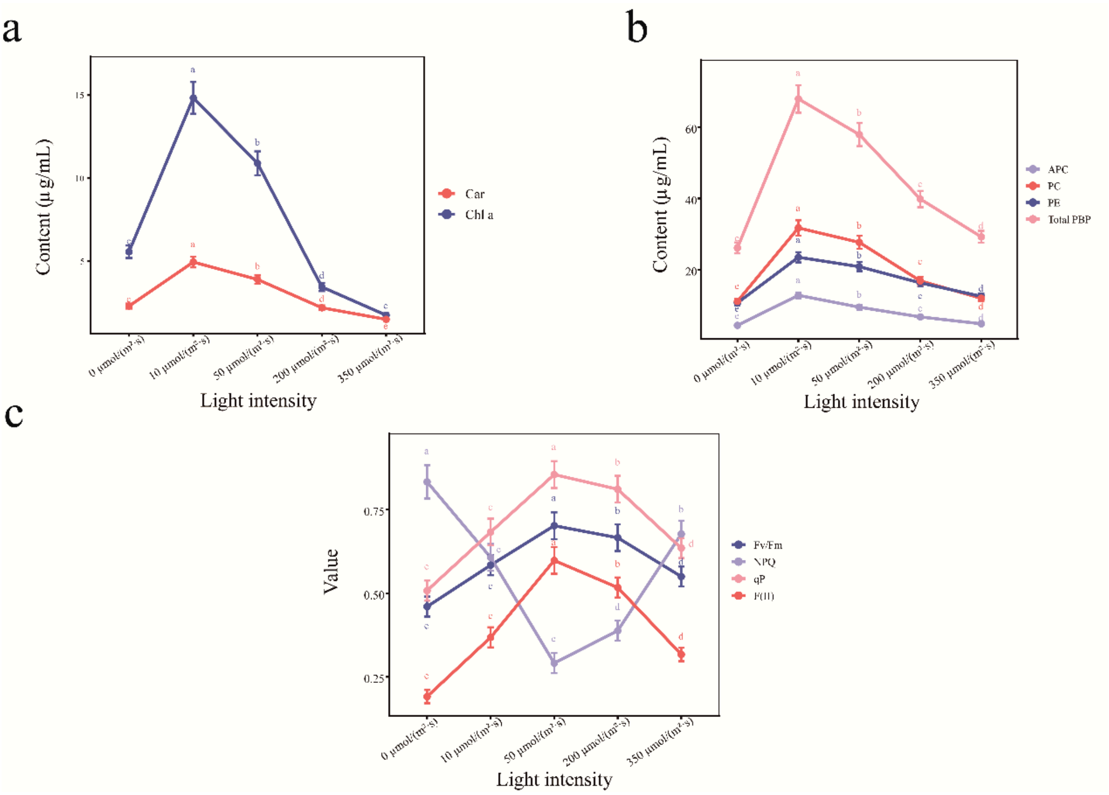

Figure S3. Physiological and phenotypic comparisons of *P. haitanensis* under different light intensity treatments. (**a**) Phycobiliprotein contents, including phycoerythrin, phycocyanin and allophycocyanin. (**b**) Photosynthetic pigment contents, including chlorophyll a and carotenoids. (**c**) Chlorophyll fluorescence parameters, including the maximum quantum yield of PSII, effective quantum yield of PSII, non-photochemical quenching and photochemical quenching. Values represent the mean, and error bars indicate SD. Different lowercase letters indicate significant differences among light intensity treatments within each parameter at $p < 0.05$, as determined by one-way ANOVA followed by Tukey's multiple comparison test.

References

1. Li, B.; Chen, C.; Xu, Y.; Ji, D..Xie, C. Validation of housekeeping genes as internal controls for studying the gene expression in *Pyropia haitanensis* (Bangiales, Rhodophyta) by quantitative real-time PCR. *Acta Oceanol. Sin.* **2014**, *33*, 152–159.

Disclaimer/Publisher's Note: The statements, opinions and data contained in all publications are solely those of the individual author(s) and contributor(s) and not of MDPI and/or the editor(s). MDPI and/or the editor(s) disclaim responsibility for any injury to people or property resulting from any ideas, methods, instructions or products referred to in the content.
